# Multimodality Imaging in Monoclonal Gammopathy of Undetermined Significance and ATTR Wild-Type Cardiac Amyloidosis

**DOI:** 10.3390/life15101493

**Published:** 2025-09-23

**Authors:** Amalia Peix, Aylen Perez, Yrving Figueredo, Leonel Torres, Lazaro O. Cabrera, Giselle Monzon, Hilda Roblejo, Alejandro Perera, Anita Brink, Diana Paez

**Affiliations:** 1 Institute of Cardiology and Cardiovascular Surgery, La Habana 10400, Cuba; aylenpb@gmail.com (A.P.); lomarcabrera.1968@gmail.com (L.O.C.); 2 Clinica Central Cira Garcia, La Habana 10400, Cuba; irvingfp2024@gmail.com; 3 Isotopes Center, La Habana 10200, Cuba; leonel.torres.cuba@gmail.com (L.T.); aperera2004@gmail.com (A.P.); 4 National Center for Medical Genetics, La Habana 10300, Cuba; giselle.monzon@infomed.sld.cu (G.M.); hilda.roblejo@infomed.sld.cu (H.R.); 5 Nuclear Medicine and Diagnostic Imaging Section, Division of Human Health, Department of Nuclear Sciences and Applications, International Atomic Energy Agency, 1400 Vienna, Austria; a.brink@iaea.org (A.B.); d.paez@iaea.org (D.P.)

**Keywords:** cardiac amyloidosis, monoclonal gammopathy of undetermined significance, technetium-99m pyrophosphate, cardiac magnetic resonance

## Abstract

Amyloidosis is characterized by the tissue deposition of insoluble fibrils derived from misfolded proteins. This case report describes a Hispanic man diagnosed with both monoclonal gammopathy of undetermined significance (MGUS) and wild-type transthyretin amyloidosis (ATTR) cardiac amyloidosis. The diagnosis was made using a combination of serological tests and multimodality cardiac imaging. The report highlights the importance of multimodality imaging in diagnosing cardiac amyloidosis, especially in cases where MGUS is also present. The patient presented with shortness of breath and was found to have cardiac abnormalities through electrocardiogram, echocardiogram, and cardiac magnetic resonance (CMR). A technetium-99m pyrophosphate (Tc-99m PYP) scan confirmed the presence of ATTR cardiac amyloidosis. Bone marrow biopsy confirmed MGUS. The patient was treated with diuretics and remained asymptomatic during follow-up. The report emphasizes the need for accurate diagnosis to differentiate between AL, ATTR, and MGUS due to their distinct clinical courses and treatments.

## 1. Introduction

Amyloidosis results from the aggregation of misfolded proteins into insoluble fibrils; the most common cardiac forms are light-chain (AL) and transthyretin (ATTR) amyloidosis [1]. Cardiac involvement typically manifests as heart failure with preserved ejection fraction, arrhythmias, and conduction disease [1]. A multidisciplinary approach is essential for diagnosing and managing cardiac amyloidosis due to its nature as a systemic condition [2].

Noninvasive imaging—speckle-tracking echocardiography, cardiac magnetic resonance (CMR), and bone-avid tracer scintigraphy (e.g., technetium-99m pyrophosphate-Tc-99m PYP)—is now central to diagnosis. In particular, Tc-99m PYP scintigraphy enables differentiation between AL and ATTR in the majority of cases [3,4].

Diagnosis of AL amyloidosis requires detection of a monoclonal protein by means of serum-free light-chain assay (SFLC), serum or urine immunofixation electrophoresis (SIFE/UIFE), serum or urine protein electrophoresis (SPEP/UPEP), or bone marrow biopsy [5]. A monoclonal paraprotein is also present in a monoclonal gammopathy of undetermined significance (MGUS), a premalignant condition characterized by a low-level monoclonal protein without the presence of a monoclonal plasma cell malignancy [6], which may coexist with ATTR and complicate diagnostic pathways. Phull et al. reported that in their sample of patients with wild-type ATTR (ATTRwt) and variant-ATTR (ATTRv), 39% in the ATTRwt cohort and 49% in the ATTR V122I cohort had an MGUS [7]. We report a Hispanic patient with an MGUS and wild-type ATTR cardiac amyloidosis.

## 2. History of Presentation

A 70-year-old Hispanic man (63 kg, 165 cm) presented with 12 months of progressive exertional dyspnea (NYHA III). He denied chest pain, palpitations, or syncope. Vital signs were stable; examination revealed mild bilateral pitting edema without jugular venous distention (JVD) or pulmonary congestion. There was no hepatomegaly. The patient’s neurological system was grossly intact.

## 3. Past Medical History

The patient was a non-smoker. Past history included well-controlled hypertension with enalapril, hyperuricemia controlled with alopurinol, bilateral carpal tunnel syndrome (six years prior), and lumbar disc herniation. Family history was notable for an uncle with heart failure.

## 4. Investigations and Management

Initial laboratory tests revealed the following: hemoglobin 13.0 g/L, hematocrit 39, erythrocyte sedimentation rate 28 mm/h, total protein 70.4 g/L, B2 microglobulin 5.5 microg/mL, albumin 42.1, creatinine 99 mmol/L, urates 490 mmol/L, total bilirubin 13 mmol/L, and direct bilirubin 4 mmol/L.

**Figure 1:**
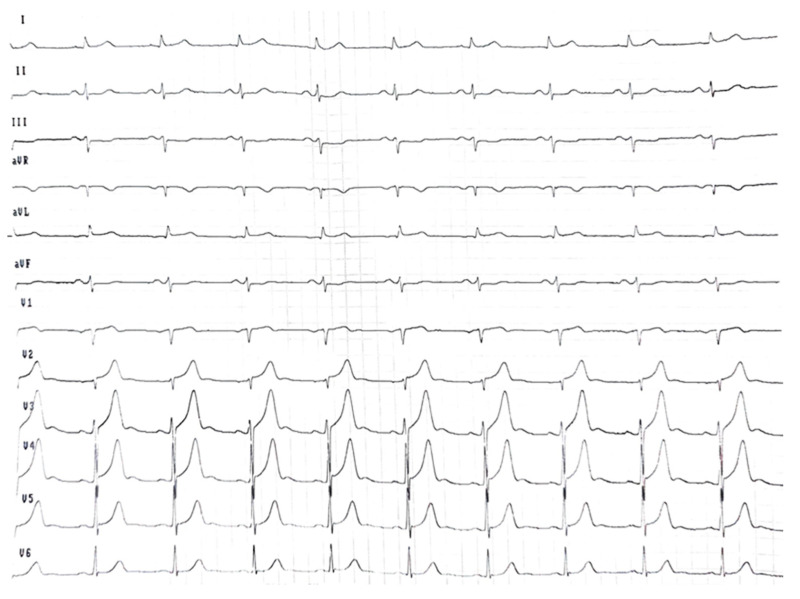
Electrocardiogram at the initial consultation showed sinus rhythm at 60 beats/min and low voltage in the limb leads.

ECG (Figure 1) showed sinus rhythm (≈60 bpm), low limb-lead voltage, and a pseudoinfarct pattern. There was no cardiomegaly and no vascular congestion on chest X-ray.

**Figure 2:**
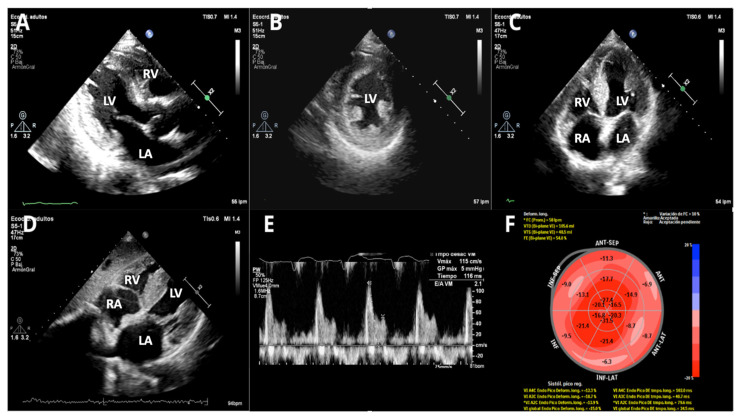
Transthoracic echocardiography (TTE). (**A**) Parasternal long axis—2D, (**B**) parasternal short axis—2D, (**C**) apical 4-chamber—2D, (**D**) subxiphoid long axis—2D, (**E**) Doppler Pulsatile wave-restrictive mitral flow, and (**F**) bull eyes, speckle tracking 2D apical myocardial sparing. It shows concentric left ventricular hypertrophy with normal left ventricular ejection fraction and grade III diastolic dysfunction. There is also right ventricular free wall thickening and moderate biatrial enlargement with interatrial septum thickening. (LA: left atrium, LV: left ventricle, RA: right atrium, RV: right ventricle).

Transthoracic echocardiography (TTE) (Figure 2) revealed concentric left ventricular hypertrophy with a maximal wall thickness of 14 mm, preserved left ventricular ejection fraction (56.8%), and grade III diastolic dysfunction. Additional findings included right ventricular free-wall thickening, biatrial enlargement with interatrial septal thickening, and a reduced global longitudinal strain (−15.4%) displaying the characteristic apical sparing pattern.

In the follow-up, diuretics were added for symptom management (furosemide 40 mg/day and spironolactone 25 mg/day).

Given the constellation of findings and carpal tunnel history, CMR, Tc-99m PYP scintigraphy, and an AL work-up were pursued.

**Figure 3:**
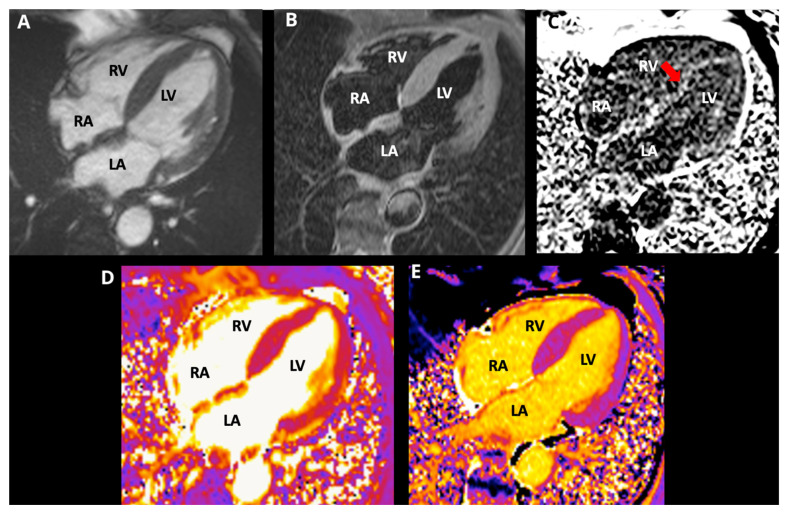
Cardiac magnetic resonance images, 4-chamber view, (**A**) cine SSFP; (**B**) T2-STIR, (**C**) PSIR; (**D**) T2 mapping; (**E**) native T1 mapping. CMR revealed symmetric LV thickening (max 17 mm), preserved biventricular systolic function (LVEF 66%, RVEF 54%), difficult myocardial nulling (red arrow), diffuse circumferential subendocardial LGE involving both ventricles with atrial/septal LGE, elevated native T1, and increased ECV (45.5%), consistent with amyloid infiltration. (LA: left atrium, LV: left ventricle, RA: right atrium, RV: right ventricle).

CMR showed mild mitral insufficiency. Thickened interatrial septum and biatrial enlargement were noted: left atrium 47.0 × 47.5 mm, area: 23.9 cm^2^, and right atrium: 50 × 43 mm, area: 19.3 cm^2^. Increased left ventricular wall thickness symmetrically involves all LV segments (maximum wall thickness: 17 mm at the medium anteroseptal). Global and regional contractility were preserved with an LVEF of 66% and right ventricular ejection fraction of 54%. A tissue tracking analysis was performed using CASIS software (QIR suite–MR version 4.1.14, Dijon, France). Longitudinal myocardial global deformation was −11.6%, circumferential global deformation was −13.7%, and radial global deformation was 42.8%. Tricuspid annular plane systolic excursion was 16.8 mm, while mitral annular plane systolic excursion was 10 mm. T2-STIR was normal. Difficult nulling of the myocardium was noted. Sequences with recovery inversion showed circumferential subendocardial transmural late gadolinium enhancement (LGE) affecting both ventricles, at the level of the basal inferolateral segment. LGE was also noted in the atria and interatrial septum. Myocardial mapping: Native T1: 1118 ms (normal value: 1024 ± 39 ms); T2: 49.4 ms (normal value: 41 (40–43] ms); extracellular volume (ECV): 45.5% (normal value: 25 ± 3%) [8]. With these CMR results, a restrictive cardiomyopathy probably due to amyloid infiltration was concluded (see Figure 3).

**Figure 4:**
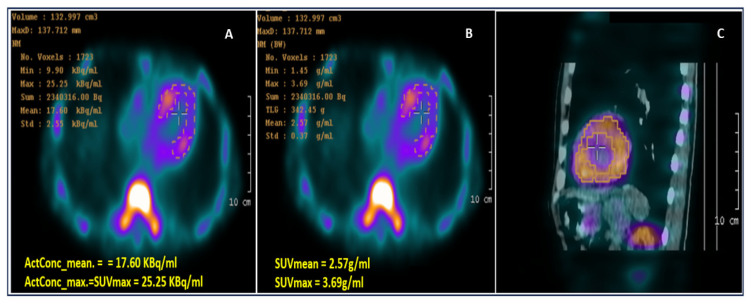
Computed 99mTc-PYP SPECT/CT images: (**A**) Activity concentration (ActCon) image; (**B**) SUV image; and (**C**) fused SPECT-CT image showing myocardium segmentation based on the thresholding method (40% of peak activity) as suggested by Caobelli F. et al. [9]. CT: computed tomography; SPECT: single-photon emission computed tomography; SUV: standardized uptake value; 99mTc-PYP: 99mTechnetium-pyrophosphate.

A Tc-99m PYP planar scan at one hour depicted defined radiopharmaceutical uptake in the cardiac area, with a heart/contralateral hemithorax index of 1.74. The SPECT image at 3 h (Figure 4) confirmed a heart-versus-ribs Perugini grade 3. There was left ventricle diffuse radiopharmaceutical uptake, without significant blood pool uptake. No significant uptake in the right ventricle or atria was noted. Based on this highly positive Tc-99m PYP scan, TTR cardiac amyloidosis was likely diagnosed, but it was recommended to differentiate it from AL amyloidosis.

**Figure 5:**
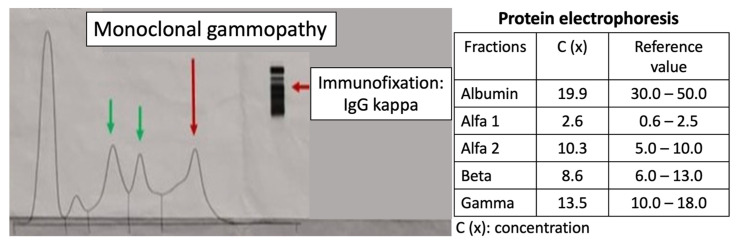
Protein electrophoresis. Presence of a monoclonal gammopathy. A decrease in albumin and a slight increase in alpha 1 and alpha 2 fractions are noted. The peaks represent (from left to right) the following: albumin, alfa-1 globulin, alfa-2 globulin, beta globulin, and gamma globulin.

**Figure 6:**
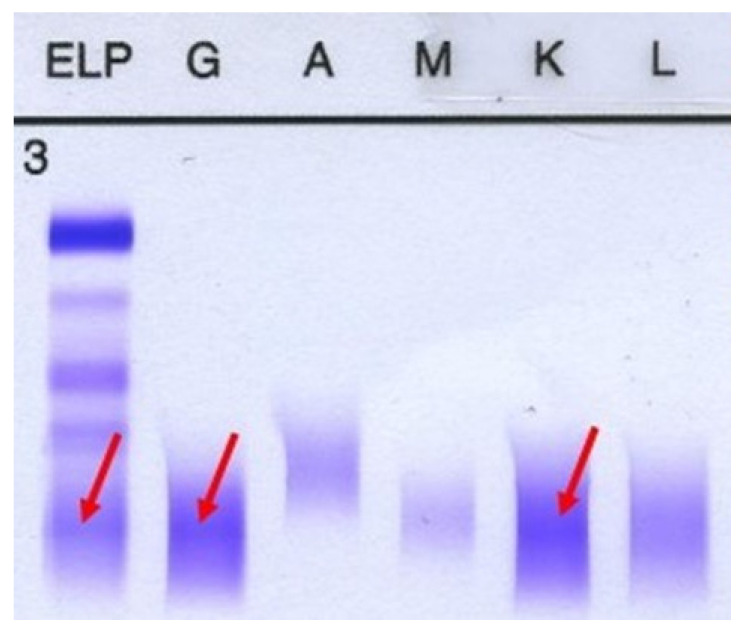
Immunofixation. A monoclonal IgG kappa component is noted. First column represents gamma globulin, the second one is IgG and the fifth column represents IgK.

**Figure 7:**
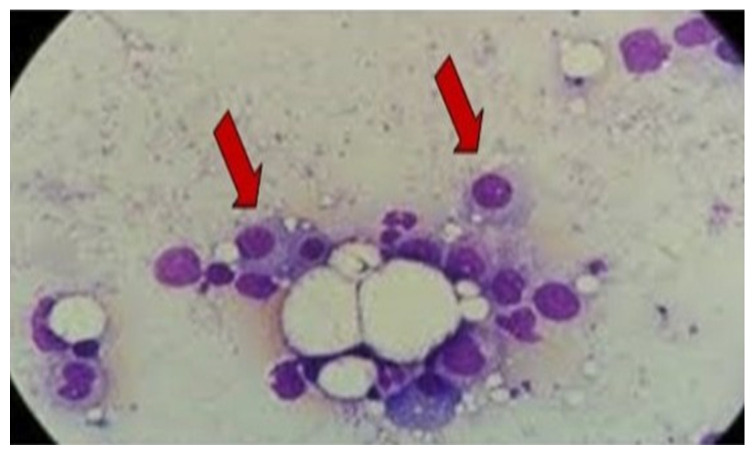
Bone marrow evaluation reported 8% well-differentiated plasma cells without infiltration, consistent with MGUS Arrows represent plasma cells. Image 100×. MGUS: monoclonal gammopathy of uncertain significance.

The protein electrophoresis shows a decrease in albumin and a slight increase in alpha 1 and alpha 2 fractions (Figure 5). A monoclonal IgG kappa component is observed in the immunofixation (Figure 6). The medulogram (Figure 7) reported the following: peripheral lamina: hypochromia x, macrocytes x. Anisopoikilocytosis, adequate leukocytes. No toxic granules. No lymphomonocytic cells. No blasts. No adequate platelets, no plasma cells in the periphery. Megakaryopoietic system: Intact. Presence of all forms of maturation. Some dysmorphic changes. Granulopoietic system: Complete. Presence of all forms of maturation. Some dysmorphic changes. Erythropoietic system: Light integer. Presence of all forms of maturation. Some dysmorphic changes. Cellularity x. Of note, 8% of plasma cells well differentiated. Blasts less than 5%. No parenchymal foreign cells. In conclusion, bone marrow not infiltrated.

The bone marrow biopsy showed the presence of the three hematopoietic series, without tumor infiltration. The definitive diagnosis of the biopsy was MGUS.

This presentation complies with the ethical standards laid down in the 1964 Declaration of Helsinki and all subsequent revisions. The review board and Ethics Committee of the Institute of Cardiology and Cardiovascular Surgery approved it, and written informed consent was obtained from the patient.

## 5. Outcome and Follow-Up

In the following months, the patient attended an outpatient follow-up visit and remained asymptomatic. A genetic study was requested, and no hereditary TTR form was detected. Specific treatment (tafamidis) was not available.

## 6. Discussion

MGUS prevalence increases with age and can coexist with ATTR, obscuring the distinction from AL when a monoclonal protein is detected. As treatments and prognosis differ among AL, ATTR, and MGUS, accurate classification is crucial. Multimodality imaging—strain echocardiography (apical sparing), CMR (diffuse subendocardial LGE, elevated native T1 and ECV), and Tc-99m PYP (grade 2–3 uptake with SPECT confirmation)—combined with targeted laboratory testing enables a noninvasive diagnosis of ATTR in most patients and helps exclude AL when monoclonal proteins represent MGUS rather than pathogenic light-chain disease. Table 1 shows the strengths and weaknesses of multimodality imaging in cardiac amyloidosis.

In patients with suspected cardiac amyloidosis, detection of a monoclonal protein may indicate either AL amyloidosis or ATTR amyloidosis coexisting with MGUS, as in the present case. MGUS is a premalignant plasma-cell disorder defined by low concentrations of a monoclonal protein without evidence of organ involvement. According to the International Myeloma Working Group, diagnostic criteria include a serum M-protein < 3 g/dL, <10% clonal plasma cells in the bone marrow, and the absence of clinical features attributable to plasma-cell proliferative disease such as hypercalcemia, renal impairment, anemia, or bone lesions. Four subtypes are recognized: IgM, non-IgM, light-chain, and secondary MGUS [6,10].

**Figure 8:**
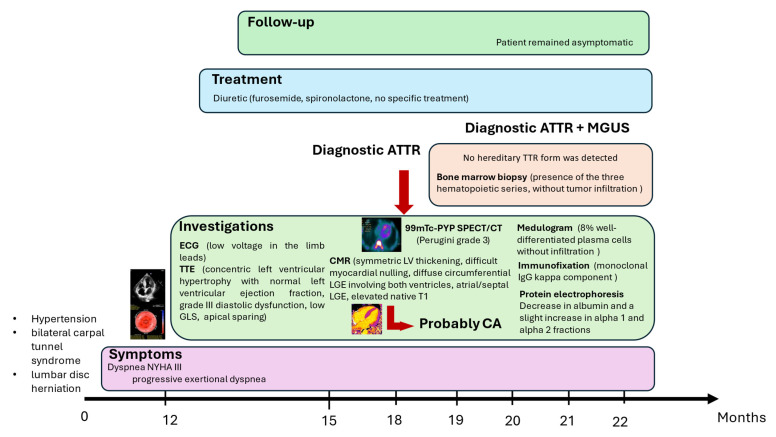
Timeline showing the clinical signs and main instrumental/laboratory findings in the patient. ATTR: transthyretin amyloidosis; MGUS: monoclonal gammopathy of uncertain significance; ECG: electrocardiogram; TTE: transthoracic echocardiogram; Tc-99m PYP: technetium-99m pyrophosphate; SPECT: single-photon emission computed tomography; CT: computed tomography; CMR: cardiac magnetic resonance; CA: cardiac amyloidosis.

Although indolent for long periods, MGUS carries a risk of progression to malignant conditions, including multiple myeloma (MM; non-IgG or IgA), Waldenström macroglobulinemia (IgM type), AL amyloidosis (IgM type), or other lymphoproliferative disorders, at an approximate rate of 1% per year [11]. No specific therapy exists for MGUS, and screening is generally not recommended in asymptomatic individuals, given its high prevalence in older populations. Evaluation is warranted only when clinical suspicion arises for MM, AL amyloidosis, WM, or other lymphoproliferative syndromes. Figure 8 presents a timeline showing the clinical signs and main instrumental/laboratory findings in our patient.

Earlier studies reported a relatively low prevalence of MGUS among patients with wild-type ATTR amyloidosis (10–18%) [12]. However, in a retrospective analysis by Phull et al., including 140 patients with biopsy-proven wild-type ATTR and 57 with V122I variant ATTR, MGUS was present in 39% and 49% of cases, respectively, diagnosed by abnormalities in serum free light-chain ratio and/or serum immunofixation [7]. These findings highlight the markedly higher prevalence of MGUS in patients with ATTR compared with the general population. In contrast, the coexistence of AL and ATTR amyloidosis within the same patient remains exceedingly rare [13,14]. Given that AL, ATTR, and MGUS differ substantially in clinical course, prognosis, and management, accurate classification and recognition of possible coexistence are essential for appropriate patient care.

## 7. Conclusions

In patients with suspected cardiac amyloidosis and a detected monoclonal protein, coexistence of MGUS and ATTR must be considered. A rigorous pathway integrating serology, bone marrow assessment, and multimodality imaging allows for precise typing—critical for prognosis and therapy. MGUS as a concomitant condition does not require therapy; however, the distinction between AL and ATTR is critical, as misdiagnosis may occur if amyloid deposits are not definitively typed in the setting of a monoclonal protein. Further research should clarify why MGUS is more prevalent among patients with ATTR than in the general population.

## Figures and Tables

**Table 1:** Strengths and Weaknesses of Multimodality Imaging in Cardiac Amyloidosis.

Modality	Strengths	Weaknesses	Differential Role (ATTR vs. AL)
Echocardiography	Widely available; detects LVH, diastolic dysfunction; apical-sparing GLS suggests amyloidosis; RV/atrial involvement visible	Limited specificity; wall thickening non-specific; cannot type amyloid	Suggests amyloidosis but cannot distinguish ATTR from AL
Cardiac Magnetic Resonance (CMR)	Tissue characterization: diffuse subendocardial LGE; quantitative mapping (↑ native T1, ↑ ECV) improves sensitivity; atrial/septal involvement	Patterns overlap among amyloid types; gadolinium limited in renal dysfunction; requires expertise/availability	Strongly supports amyloidosis but does not definitively type ATTR vs. AL
Tc-99m PYP scintigraphy	High PPV for ATTR when grade 2–3 myocardial uptake presents with SPECT confirmation; enables noninvasive diagnosis; H/CL ratio adds confidence	False positives if only planar imaging; rare uptake in AL; requires exclusion of pathogenic monoclonal protein	Primary noninvasive discriminator: grade 2–3 uptake + negative AL work-up strongly supports ATTR

LVH: left ventricular hypertrophy; GLS: global longitudinal strain; RV: right ventricle; LGE: late gadolinium enhancement; ECV: extracellular volume; PPV: Positive Predictive Value; PYP: pyrophosphate; H: heart; CL: contralateral lung.

## Data Availability

The data presented in this study are available on request from the corresponding author due to (privacy and ethical reasons).

## Abbreviations

AL	light-chain amyloidosis
ATTR	transthyretin amyloidosis
CMR	cardiac magnetic resonance
ECG	Electrocardiogram
ECV	extracellular volume
GLS	global longitudinal strain
LGE	late gadolinium enhancement
LVEF	left ventricular ejection fraction
LVH	left ventricular hypertrophy
MGUS	monoclonal gammopathy of undetermined significance
MM	multiple myeloma
SFLC	serum-free light-chain assay
SIFE/UIFE	serum or urine immunofixation electrophoresis
SPEP/UPEP	serum or urine protein electrophoresis
Tc-99m—PYP	technetium-99m-Tc99m-pyrophosphate
TTE	transthoracic echocardiogram
WM	Waldenström’s Macroglobulinemia
